# Congenital bronchopulmonary foregut malformation: systematic review of the literature

**DOI:** 10.1186/s12887-019-1686-1

**Published:** 2019-09-02

**Authors:** Gang Yang, Lina Chen, Chang Xu, Miao Yuan, Yuan Li

**Affiliations:** 1 Department of Pediatric Surgery, West China Hospital, Sichuan University, Guoxue Xiang No.37, Chengdu, 610041 China; 2 Department of Pediatrics, West China Second University Hospital, Sichuan University, Chengdu, 610041 Sichuan China

**Keywords:** Congenital bronchopulmonary foregut malformation, Esophageal bronchus, Esophageal lung, Systematic review

## Abstract

**Background:**

Congenital bronchopulmonary foregut malformation (CBPFM) is a rare congenital malformation involving both the digestive and respiratory system. Early diagnosis is difficult, and delayed recognition may result in considerable complications. The aim of the study was to identify and analyze the clinical characteristics and radiological features of CBPFMs.

**Methods:**

A systematic review was conducted in accordance with PRISMA guidelines. PubMed, Ovid database, EMBASE were searched for relevant publications to identify all published case-reports of CBPFM since 1992. Data about the demography, clinical presentation, pathology, imaging features, treatment and prognosis were collected.

**Results:**

Sixty-one cases were included in our study. Cases were aged from 1 day to 59 years with the majority aged 3 years or younger. The most common type was group III (37.7%), followed by group II (29.5%)group I (27.9%) and group IV (4.9%). The presentations included respiratory distress (32.8%), cough/choking following food intake and other presentations associated respiratory infection. Thirty-eight cases (62.3%) were diagnosed by upper gastrointestinal series (UGI). Misdiagnosis was common. Eight cases (13.1%) of the included cases died.

**Conclusions:**

Early recognition and extensive delineation of the anatomy of CBPFM are important to correct these anomalies successfully. UGI is the first choice to confirm the abnormal bronchus communicating with the esophagus. Resection of abnormal pulmonary tissue, lobe or even unilateral lung is preferred. Reconstruction procedures are feasible in selected patients.

**Electronic supplementary material:**

The online version of this article (10.1186/s12887-019-1686-1) contains supplementary material, which is available to authorized users.

## Background

Communicating bronchopulmonary foregut malformation (CBPFM) is a rare congenital anomaly defined by a patent congenital communication between the esophagus or stomach and an isolated portion of the respiratory tract. Lesions including a lobar bronchus arising from the esophagus are described as an esophageal bronchus. If the main bronchus originates from the esophagus, it is termed esophageal lung [1]. In 1992, based upon the occurrence of related defects and the level of the communication, Srikanth et al. reviewed 57 cases and proposed a system to classify CBPFM into four groups [2]. Group I is associated with esophageal atresia and tracheoesophageal fistula and contains 2 subdivisions, that is, group IA, total sequestered lung communicating with the foregut, and group IB, Sequestered anatomic lobe or segment communicating with the foregut. Group II is characterized by the absence of a mainstem bronchus arising from the trachea and the total sequestered lung (usually the right) communicating with the lower esophagus. Group III occurs when an isolated part of the lung is communicating with the esophagus and group IV occurs when there is communication between a normal bronchial system and the esophagus.

Early diagnosis of CBPFM is difficult for the rarity of this entity and the nonspecific symptoms. We presented an infant with CBPFM and strictly reviewed the available literatures since Srikanth’s classification proposed to describe the demography, clinical presentation, pathology, imaging features, treatment and prognosis of CBPFM.

## Methods

### Case

**Fig. 1:**
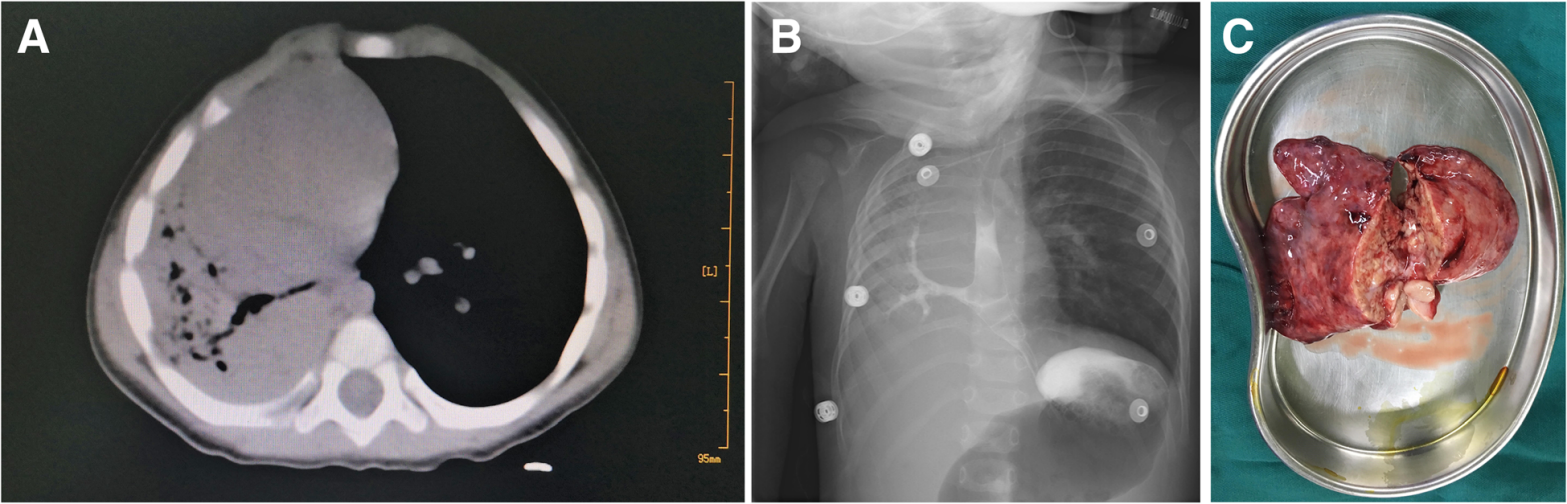
**a** CT scan showed hypoplasia of the right lung and the right mainstem bronchus originating from the distal esophagus. **b** The UGI revealed filling of the right main bronchus from the esophagus with opacification of the bronchial tree on the right side. **c** Diffuse abscesses were formed in the right lung parenchyma

A 3.4 kg female baby was born after an uneventful pregnancy. After birth, she presented with mild respiratory distress and chest infection. A computed tomography (CT) scan was performed, which showed hypoplasia of the right lung and a dextrocardia. Antibiotics were administered, and the symptoms alleviated. After that, the baby repeatedly presented with recurrent episodes of fever, cough, tachypnea and retraction which were controlled and managed with oral antibiotics. At 6 months old, she was admitted for persistent high fever (40 °C) and dyspnea. CT scan showed hypoplasia of the right lung with multiple air bronchograms. The right mainstem bronchus originated from the distal esophagus and coursed to the right lung (Fig. 1A). The upper gastrointestinal series (UGI) using iohexol (Omnipaque 350, GE Healthcare, Shanghai, China) was performed to rule out the possibility of H-type tracheoesophageal fistula (TEF). It revealed filling of the right main bronchus and bronchial tree, which directly originated from the esophagus (Fig. 1B). Bronchoscopy revealed a blind ended right bronchial stump and thus, the diagnosis of CBPFM (group II) was established. Echocardiography showed a small patent ductus arteriosus with good biventricular function and the left pulmonary artery arising distally from the right pulmonary artery.

Intra-operative findings revealed a heavily consolidated hypoplastic right lung with a single thin right pulmonary artery and single right pulmonary vein. Diffuse abscesses were formed in the lung parenchyma (Fig. 1C). The bronchus to the lung arose from the lower third of the esophagus. Right pneumonectomy with resection of esophageal bronchus was performed, and the abnormal opening in the esophagus was repaired. Postoperative recovery was uneventful, and the temperature returned to normal on the second day after surgery. A second UGI of the entire esophagus did not detect any leaking or fistula. The patient was discharged on postoperative day 7. She was doing well on follow-up 6 months later.

### Systematic review

For reviewing of the literature, we conducted the systematic review following the recommendations of the Preferred Reporting Items for Systematic Reviews and Meta-Analyses (PRISMA) statement [3]. PubMed, OVID database and EMBASE were systematically searched for relevant articles published in English from January 1992 to August 2018. The search terms/syntax in PubMed was as follows: (bronchopulmonary foregut malformation OR “esophageal bronchus” OR “esophageal lung”). The titles and abstracts of all potentially relevant articles were read to determine their relevance. Full articles were also scrutinized if the title and abstract were unclear. Reference lists of identified articles were screened to ensure completeness of the search. All identified articles were independently assessed by two authors. Detailed data regarding patient characteristics, symptoms, initial diagnoses, diagnostic work-up, treatment and outcome were extracted into an electronic data sheet in a standardized manner.

The inclusion criteria were as follows: (I) Original studies describing individual cases of CBPFM in accordance with the definition proposed by Srikanth. (II) Studies describing adequate clinically relevant details. The exclusion criteria were as follows: (I) The malformation did not comply with the pattern of CBPFM. (II) Individual cases previously described in another study. Review articles and editorials were excluded. No statistical analysis was used for this review.

## Results

**Fig. 2:**
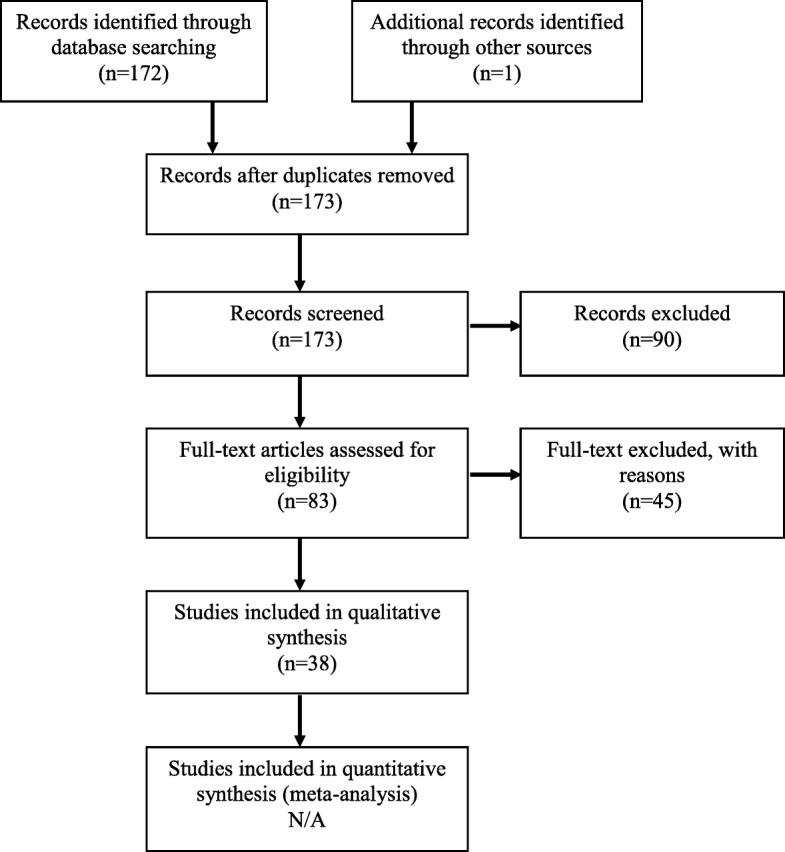
Preferred reporting items for systematic reviews and meta-analyses flowchart

One hundred and seventy-two papers were identified by searching the databases, one additional article was identified by manual searching. No reports were repetitive. Eighty-three reports were eligible for full-text screening. Of them, 38 papers matched our criteria and were included [2, 4–40]. The selection study process is showed in the PRISMA flowchart in Fig. 2. They included 60 cases of CBPFM. The 134 papers excluded were review articles, animal cases, published in a language other than English or malformation not meeting the diagnostic criteria depicted by Srikanth et al. Therefore, 61 cases were reviewed including our case (Table 1). The table in the Additional file 1: Table S1 shows the details of the included cases.

**Table 1:** Clinical characteristics of included patients in the systematic review

Clinical Features	Group					Total (*n* = 61)
	IA (*n* = 13)	IB (*n* = 4)	II (*n* = 18)	III (*n* = 23)	IV (*n* = 3)	
Sex						
Male	7 (53.8%)	2 (50.0%)	8 (44.4%)	9 (39.1%)		26 (42.6%)
Female	6 (46.2%)	2 (50.0%)	10 (55.6%)	9 (39.1%)	3 (100%)	30 (49.2%)
Not stated				5 (21.7%)		5 (8.2%)
Age						
Newborn	13 (100%)	3 (75.0%)	10 (55.6%)	12 (52.2%)		38 (62.3%)
Infant (< 1 yr)			5 (27.8%)	2 (8.7%)	1 (33.3%)	8 (13.1%)
Child (< 18 yr)		1 (25.0%)	3 (16.7%)	3 (13.0%)	1 (33.3%)	8 (13.1%)
Adult				6 (26.1%)	1 (33.3%)	7 (11.5%)
Not Stated			1 (5.6%)			1 (1.6%)
Side						
Right	9 (69.2%)	4 (100%)	14 (77.8%)	14 (60.9%)	3 (100%)	44 (72.1%)
Left	3 (23.1%)		4 (22.2%)	7 (30.4%)		14 (23.0%)
Bilateral				2 (8.7%)		2 (3.3%)
Not stated	1 (7.7%)					1 (1.6%)
Arterial supply						
Systemic	2 (15.4%)	1 (25.0%)	2 (11.1%)	17 (73.9%)		22 (36.1%)
Pulmonary	3 (23.1%)		11 (61.1%)	2 (8.7%)	1 (33.3%)	17 (27.9%)
Not stated	8 (61.5%)	3 (75.0%)	5 (27.8%)	4 17.4%)	2 (66.7%)	22 (36.1%)
Divagnostic method						
UGI	10 (76.9%)	2 (50.0%)	14 (77.8%)	10 (43.5%)	2 (66.7%)	38 (62.3%)
CT	2 (15.4%)	1 (25.0%)	4 (22.2%)			7 (11.5%)
Bronchoscopy	1 (7.7%)					1 (1.6%)
Intraoperative		1 (25.0%)		7 (30.4%)		8 (13.1%)
Other				6 (26.1%)	1 (33.3%)	7 (11.5%)
Outcome						
Survive	7 (53.8%)	2 (50.0%)	12 (66.7%)	14 (60.9%)	3 (100%)	38 (62.3%)
Death	4 (30.8%)	2 (50.0%)	1 (5.6%)	1 (4.3%)	0 (0)	8 (13.1%)
Not stated	2 (15.4%)		5 (27.8%)	8 (34.8%)		15 (24.6%)

*UGI*, upper gastrointestinal series

### Demographic characteristics

The gender of five cases was not reported, there were 26 males and 30 females, and male to female ratio was 0.87:1. The sex distribution among each CBPFM group was similar. The age of cases ranged from 1 day to 59 years. A large proportion of these patients were aged 1 year or younger at presentation (*n* = 46) and 38 presented immediately after birth. Seven adults have been described.

### Classification and affected side

Among the included 61 cases, there were 13 cases (21.3%) in group IA, 4 cases (6.6%) in group IB, 18 cases (29.5%) in group II, 23 cases (37.7%) in group III and 3 (4.9%) cases in group IV. Most of the malformations located in the right side (72.1% vs 23.0%). Two cases (3.3%) had bilateral malformations. The right to left ratio was 3.1:1. The blood supply of abnormal lung tissue was reported in 39 cases, with supply from the pulmonary artery in 17 cases and the systemic artery in 22 cases.

### Gestation and family history

Polyhydramnios was reported in 10 cases (3 cases in Group I, 3 cases in Group II, 3 cases in Group III and 1 case in Group IV). Fetal MRI detected the tubular structure directing to the gastroesophageal junction during the second-trimester in 5 cases. These 5 cases belonged to Group III. A mass was detected in the fetal chest cavity in 2 cases.

There were four cases from three sets of twins. Two cases were monochorionic, monoamniotic twins. One twin of was classified in group IA with VACTERL association with the twin sibling in group II without associated malformations. The other two cases were twins whose siblings were not affected.

### Symptoms

Twenty patients presented with respiratory distress after birth. Patients with group I malformations presented with drooling, feeding intolerance and failure to pass nasogastric tube because of esophageal atresia. Recurrent respiratory infection, presenting with cough and/or fever, were the main symptoms in older children and adults (10/22, 45.5%). Other symptoms included cough/choking following food intake (*n* = 5), hemoptysis (*n* = 2), nocturnal cough (*n* = 1), epigastric pain (*n* = 1).

### Associated malformations

Since CBPFM group I is associated with esophageal atresia and tracheoesophageal fistula (EA/TEF), EA/TEF was not included as an associated malformation in our review. Cardiovascular anomalies were the most common associated malformation (*n* = 11, 18.0%), followed by VACTERL association (*n* = 6, 9.8%), skeletal malformation (*n* = 6, 9.8%), anorectal malformation (*n* = 2, 3.3%), diaphragmatic hernia (*n* = 2, 3.3%) and other less frequently malformations.

### Diagnosis

For group I, chest radiographs were obtained in 70.6%. The hazy hemithorax and mediastinal shift with dextrocardia were revealed in 52.9%, hypoaerated or normal lung in 17.6%. Only one case of CBPFM was suggested by CT scan initially. One case without chest X-ray was demonstrated during surgery for EA/TEF. Fourteen cases (82.4%) with group I CBPFM were misdiagnosed and were initially operated on for EA/TEF (ligation of tracheoesophageal fistula, primarily esophageal anastomosis or gastrostomy). Definitive diagnoses were confirmed by further evaluations that were prompted by persistent atelectasis of one lung, refractory respiratory distress or routine postoperative UGI.

Opacification of ipsilateral lung and mediastinal shift were also the uniform presentations in chest radiograph of group II (100%). Plain X-ray in group III and group IV were reported in 42.3%. The presentations were various, including mediastinal or pulmonary mass (19.2%), consolidation of part of the lung (23.1%).

The diagnoses of CBPFM were confirmed by UGI in 38 case (62.3%), CT in 7 cases (11.5%), bronchoscopy in 1 case (1.6%) and intraoperative finding in 8 cases (13.1%). There were five cases (8.2%) diagnosed prenatally by ultrasonography and fetal MRI.

### Treatment

Fifty-five cases underwent surgeries. Unilateral pneumonectomies were performed in six cases with group I, 11 cases with group II and one case with group IV. Three cases with Group IA and four cases with group II received reimplantation of the esophageal bronchus. Lobectomies or resection of aberrant lung tissue and bronchus were performed in 24 cases with group IB, group III or group IV. The procedures of the other 6 cases were unknown.

### Outcome

Eight patients died in this series with 4 in group I, 2 in group IB, 1 in group II and group III respectively. However, the outcomes of 15 cases (24.6%) were unknown, so the mortality was 17.4% in 45 cases with outcome reported. In survivors, 44.3% were reported as uneventful follow-up. Eight cases (13.1%) had respiratory morbidities such as air way stenosis, tracheomalacia, recurrent respiratory infection or difficulty in weaning from ventilator. Postpneumonectomy syndrome occurred in one case.

In these series, seven cases received bronchial reconstruction procedures. The longest follow-up was 7 years. Two cases died (one during operation, the other on postoperative day 5). Three cases received more more than one operation for tracheal stenosis, bronchial stenosis, feeding difficulty, bronchomalacia, accompanied laryngotracheoesophageal cleft. The other complications included severe gastroesophageal reflux, anastomosis stricture, recurrent respiratory infection. Only two cases were reported as free of symptoms with normal growth in long-term follow-up.

## Discussion

The term congenital bronchopulmonary foregut malformation was proposed by Gerle et al. in 1968 to describe either an intralobar or an extralobar sequestration communicating with the gastrointestinal tract [41]. Srikanth et al. proposed the classification based on reviewing 57 cases of CBPFM [2].

The embryogenesis of CBPFMs is not clear though it was treated as a sub-type of bronchopulmonary foregut malformation including foregut vascular abnormalities, lung parenchymal abnormalities and airway anomalies [42]. This malformation was proposed to occur when there was a congenital communication between the lung and foregut because of a focal mesodermal defect. The aberrant lung tissue was separated with the rest of the lung and trachea during the rapid elongation of the esophagus, which accounted for the missing portion of the corresponding bronchial tree [27]. CBPFM also occurred in an adriamycin-induced rat model of esophageal atresia. The observations were consistent with the hypothesis that CBPFM and EA were variations of the same spectrum of abnormalities and may have a similar etiology [43].

Our series were in agreement with that reported by Srikanth et al. in the female and right-side preponderance. The distribution of numbers among groups was similar. But the mortality in Srikanth’s series was 24.6% compared with 17.4% after 1992. The increased survival rate may be attributed to improvements in critical care management of affected newborns. Nevertheless, delay of diagnosis was still common for the rarity of the entity. Especially for group I, the existence of CBPFM is obscured by the presentation of EA/TEF. Though the abnormal pulmonary tissue could be aerated through the communication of the esophagus before the inflammation commenced, collapse of the ipsilateral lung was the typical presentation. Hence the persistent or worsening unilateral lung collapse with or without EA/TEF should raise the suspicion and warrant further examinations to exclude CBPFM. UGI was the first choice to delineate the bronchial tree connected to the esophagus, but the value was limited in group I due to atresia of the proximal esophagus. In this circumstance, bronchoscopy should be considered. Computed tomography (CT) is helpful in evaluating the pulmonary damage and vascularization.

Prenatal diagnosis was possible in some high-volume institutions by experienced radiologist [20]. In fetal MRI, a tubular T2 hyperintense structure (bronchocele) directed from the lung lesion to the gastroesophageal junction can be seen. The congenital lung lesions are now more commonly recognized on screening anatomic ultrasound, and CBPFM should be considered as a differential diagnosis.

No predisposing factors have been identified in the etiology of CBPFM, although the observed concordance of this lesion in a pair of twins argues in favor of a possible underlying genetic predisposition. Because the frequency of the associated malformations is high, preoperative screening examinations was important. Echocardiography is mandatory preoperatively as cardiovascular malformations were found in 18.0% of the cohort of patients. VACTERL association was not uncommon and a comprehensive examination should be performed. Accompanying airway stenosis was the most difficult problem which always resulted in long-term morbidity. The management requires extensive experience.

Surgery was the only method to effectively manage this malformation. Conventional treatment is resection of the hypoplastic lung which has been destroyed by recurrent infection. Pneumonectomy was performed in most patients with group IA or group II. Unilateral lung resection in neonates and infants was well tolerated. However, the prevalence of long-term consequences, such as chest wall deformation, scoliosis and postpneumonectomy syndrome were unclear. One patient developed postpneumonectomy syndrome in our review. A tissue expander was inserted, and the symptoms subsided with growth. The other choice was reconstruction of the esophageal bronchus. This was the theoretically preferential procedure to preserve the affected lung and prevent the complications of pneumonectomy. The first successful reconstruction effort was reported by Michael et al. in 1997 [16]. Seven reconstruction procedures were included in our study with one simultaneously undergoing pulmonary artery reimplantation. The results were dismal due to the airway stenosis or accompanying malformations. Four out of the 5 surviving cases presented with varying degrees of airway stenosis which required surgical corrections or dilatations. The results of reported reconstruction surgery were unsatisfactory. Therefore, attempts to reconstruct the bronchus should be confined to stable patients with non-infectious lung after exclusion of significant associated malformations.

There are some limitations in our study. Firstly, selection bias cannot be ignored due to the possibility that more successful interventions were published. Nevertheless, our study samples may be representative because the distribution of sex, classifications and affected side were similar to previously reported studies. Secondly, the outcomes of 15 cases included were not reported, and these cases were excluded from calculated mortality. The lack of adequate cases and information may result in inaccurate mortality rate.

## Conclusion

CBFPM is rare and the outcome depends on the associated malformations and early recognition. Persistent opaque hemithorax with ipsilateral mediastinal shift on chest radiograph should raise the suspicion of CBFPM in infants. UGI is the preferred method to delineate the abnormal bronchus communicating with the esophagus. CT and bronchoscopy are also valuable in diagnosis or screening for accompanying malformations. Echocardiography is mandatory due to the high frequency of associated congenital heart disease. Resection of abnormal pulmonary tissue, lobe and even unilateral lung is the reasonable treatment in most cases. The decision of parenchymal sparing surgery, such as reimplantation of bronchus, should be taken cautiously due to the high possibility of long-term complications.

## Additional file

Additional file 1: **Table S1.** Characteristics of included studies. (DOCX 52 kb)

## Data Availability

All data generated or analyzed during this study are included in this published article and its supplementary information files.

## Abbreviations

CBPFM: Congenital bronchopulmonary foregut malformation
EA/TEF: Esophageal atresia and tracheoesophageal fistula
UGI: Upper gastrointestinal series
