# Supplementary material for: Congenital bronchopulmonary foregut malformation: systematic review of the literature
Source: BMC Pediatr. 2019 Sep 2;19:305. doi: 10.1186/s12887-019-1686-1 (PMC6721191; doi:10.1186/s12887-019-1686-1)
Supplement: Supplementary file 1 — Table S1. Characteristics of included studies. (DOCX 52 kb) [file 12887_2019_1686_MOESM1_ESM.docx]

Additional file 1. Characteristics of included studies

|  | **Age of presentation** | **Gender** | **Presentation** | **Associated malformation** | **Side** | **blood supply** | **X-ray** | **CT** | **Constrast** | **Bronchoscopy** | **Other** | **Group** | **Treatment** | **Comfirm** | **outcome** |
| --- | --- | --- | --- | --- | --- | --- | --- | --- | --- | --- | --- | --- | --- | --- | --- |
| **Alsaadi (2018)** | 3 days | M | Dyspnea | ARM, VSD, PDA, left pulmonary sling | Right |  | initial chest radiograph was normal. Subsequent right lung collapse, compensatory overinflated left lung | CT: right main bronchus agenesis, anomalous origin of distal part of the esophagus near GE junction. | reflux of contrast through the anomalous airway communication with the lower part of the esophagus | no |  | II | unknown | CT， contrast |  |
| **Ren (2017)** | 4 hours | M | moaning, feeding intolerance, vomiting, cyanosis, RDS | ASD, PDA | Left （LLL） | systematic: intercostal artery |  | blood supply originating from systemic circulation, soft tissue density shadows in the posterior basal segment of the left lower lung lobe | congenital bronchopulmonary foregut malformation | no |  | III | Lobectomy | contrast | half a year, normal |
| **Ren (2017)** | 1 d | M | RDS | acleistocardia | Right |  |  |  | abnormal passage in the esophagus |  |  | IB | resection the abnormal tissue | contrast | Dead |
| **Colleran (2016)** | 14 m | F | chronic cough, aspiration, inability to swallow solid, failure to thrive | VACTERL | right apical lower lobe |  |  | atelectasis and consolidation, esophageal bronchus | TEF，contrast agent extending from the esophgus |  |  | IB | Surgery | Contrast | Doing well |
|  | Newborn | M |  |  | right | systematic: aorta | Some aeration RLL at 24 h, white-out of right lung | atelectasis and consolidation, hypo plastic right pulmonary artery | CT findings were specific, not undergo upper GI series prior to surgery |  |  | IA | Surgery | CT | Doing well |
|  | Newborn | F |  | VACTERL | right |  | White-out of right lung | atelectasis and consolidation | esphageal bronchus |  |  | II | Surgery | CT, contrast | Doing well |
|  | Newborn | F |  | VACTERL | right |  | White out of right lung | atelectasis and consolidation | solitary right-side bronchus arise from distal esophagus |  |  | II | Surgery | cotrast | Doing well |
|  | Newborn | M |  | VACTERL | right |  | Hypoplatic lung, dextrocardia | atelectasis and consolidation | esophageal bronchus, arising from the right-side distal esophagus |  |  | IB | Surgery | CT, contrast | Doing well |
| **Kim (2016)** | 59 years old | F | Hemoptysis and frequent lower respiratory infection |  | left | systematic | No | Intralobar sequestration in LLL | No |  |  | III | lobectomy | intraoperative | Well |
| **Patil (2016）** | 1 m | F | Severe RDS and chest infection |  | right | pulmonary A. | opacification of the right lung with reduced lung volume, air bronchograms, and mild compensatory hyperinflation of the left lung | hypoplasia of the right lung with multiple air bronchograms.Only left mainstem bronchus was visualized.The right mainstem bronchus originated from distal esophagus | opacifying the bronchial tree on the right side |  |  | II | Right pneumonectomy | CT, contrast | Well |
| **Partridge (2015)** | G21+6 weeks | F |  |  | right (RLL) | systematic |  | lung mass, multiple systemic feeding arteries. esophgeal bronchus was not visualized by CT or esophageal | esophageal bronchus was not shown |  | prenatal US and fMRI: right lung mass, tubular structure extending to the GE junction | III | right lower lobectomy | prenatal US, fMRI | 2 m: well, normal growth |
|  | G23 weeks |  |  |  | Left (LLL) | systematic |  | esophagus bronchus not visulized |  |  | fMRI: tubular T2 hyperintense structure from the lung to the GE junction | III | resection |  | no concern |
|  | G21 weeks |  |  |  | Left (LLL) | systematic |  | esophageal bronchus not visualized |  |  | fMRI: tubular T2 hyperintense structure from the lung to the GE junction | III | resection |  | no concern |
|  | G20 weeks |  |  |  | Left (LLL) | systematic |  | esophageal bronchus not visualized |  |  | fMRI: tubular T2 hyperintense structure from the lung to the GE junction | III | resection |  | no concern |
|  | G22 weeks |  |  |  | Right (RLL) | systematic |  | RLL hyperlucent lesion, esophageal bronchus not visualized |  |  | fMRI: tubular T2 hyperintense structure from the lung to the GE junction | III | resection |  | no concern |
| **Bokka (2015)** | 2 years old | F | recurrent lower respiratory tract infection, choking following food intake, fever, cough, RDS |  | right | pulmonary | hazy right hemithorax with mediastinal shift to the right side. | right lung hypoplasia with total collapse | filling of right main bronchus directly from the esophagus | Blind ended right bronchial stump |  | II | Right pneumonectomy | contrast | well |
| **He (2015）** | newborn | M | RDS,atelectasis of the right lung |  | right |  | EA-TEF, dextrocardia, atelectasis of the right lung, hyperinflation of the left lung | atelectasis of the right lung, agenesis of the right main bronchus and stenosis of the lower trachea and left main bronchus | postop: right main bronchus arose from the lower esophagus |  |  | IA | fistula occlusion | contrast | Died at 5 month, ARDS |
|  | Newborn | M | SGA，unable to swallow his saliva，dyspnea |  | right | pulmonary | dextrocardia, atelectasis of the right lung, hyperinflation of the left lung | Postop: nonaerated hypoplastic right lung, agenesis of the right main bronchus, no connection between the esophagus and the right lung or trachea were observed | postop: continuity between the lower esophagus and right lung | no bifurcation of the trachea or ostium to the right bronchus |  | IA | right pneumonectomy | contrast | 9 months, well |
| **Chung (2013)** | Newborn | F | Cyanosis, chest retraction, tachypnea | PDA, ASD |  | pulmonary | hazy right hemithorax, mediastinal shift to the right side, patchy opacity at the right lung base due to the partially collapsed hypoplastic lung (aerated right lung) | postop: mediastinal shift to the right with the absence of an aerated right upper lung | postop: right main bronchus arising from the lower esophagus, |  |  | IA | right pneumonectomy | contrast | 3 mon, well |
| **Park (2012)** | 23 months | M | Recurrent pneumonia |  | right (RUL) |  |  | right upper lobe bronchus originated from the esophagus, the right upper lobe was collapsed, consolidated, bronchiectatic changes | right upper lobar bronchus originated from the esophagus |  |  | III | Thoracoscopy right upper lobectomy | contrast | 6 months well |
| **Takamizawa (2012)** | Newborn | M | RDS | deformity of the thoracic vertebra， fusion of the ribs; complete tracheal rings; Congenital tracheal stenosis | right | pulmonary | airless right lung; aeration of the right lung on 5th days | a right main bronchus arising from the lower esophagus | a right main bronchus arising from the lower esophagus | complete tracheal rings |  | II | 5 months: reconstruction | contrast, CT | tracheal stenosiis |
| **Boersma (2012)** | Newborn | M | SGA, unable to swallow saliva. dyspnea, persisting tachypnea, bypercapnea | right-sided hypoplastic pulmonary arteries, pulmonary hypertension； tracheomalacia. | right |  | dextrocardia, atelectasis of the right lung and compensatory hyperinflation of the left lung. EA-TEF. persisting opacification of the right lung | postop: non-aerated hypoplastic right lung. The air bronchogram of the right main stem bronchus showed continuity to the lower esophagus |  | postop: No bifurcation of the trachea to the right bronchus. esophago-gastroscopy: a small fistula was seen in the lower esophagus |  | IA | right pneumonectomy | CT, bronchoscopy | 10month：postpneumonectomy syndrome. recurrent upper and lower airway infections |
| **Katayama （2010）** | 20 years olds | F |  |  | right | systematic | abnormal mediastinal shadow | mediastinal mass |  |  |  | III | resection | intraoperative findings | uneventful |
| **Sugandhi (2011)** | 8 months | M | Intermittent fever, RDS |  | right | pulmonary | hazy right hemithorax with mediastinal shift to right side | right lung hypoplasia with total collapse of right lung | filling of right main bronchus directly from the esophagus | blind ending right bronchial stump |  | II | Right pneumonectomy | contrast, bronchoscopy | 2 years， mild scoliosis |
|  | 3 years old | F | Recurrent respiratory tract infection, productive cough and fever, coughing and vomiting after feeds |  | right | pulmonary | collapsed right lung with mediastinal shift to right | collapse and consolidation of the right lung, a dilated lower esophagus | esophagus entering the right lower part of the collapsed lung |  |  | IV | Right pneumonectomy | contrast | 3 months, normal |
| **Katz (2010)** | 4 months | M | cough worse with feeding，fever |  | left | pulmonary | diffuse infiltrative pattern of the left lung field. | poorly aerated abnormal left lung, suspicion for a bronchopulmonary fistula. | left main stem bronchus originated entirely from the distal esophagus | blind ending left main stem bronchus |  | II | thoracoscopic pneumonectomy | Contrast, CT suspicion | 14 months, doing well |
| **Matsusaka (2010)** | 43 yrs old | M | dysphagia |  | right |  |  | circumferentially thickened wall at the stenotic part | narrowing at the lower esophagus |  | upper GI endoscopy: stenosis in the lower esophagus; biopsy: squamous cell carcinoma | III | chemoradiotherapy, resection | intraoperative, pathology |  |
| **Nakaoka (2009)** |  | M | severe dyspnea, | cleft lip and palate. laryngotracheoesophageal cleft (type III) | left | systematic | poor lung permeability, air in the alimentary tract, and a coil up sign | right aortic arch with the left pulmonary artery originating from the ascending aorta | Through G-tube. TEF at the tracheal bifurcation and the left main bronchus branched from the lower esophagus |  |  | IA | reconstruction | contrast | 3 years old , growth retarded. |
| **Verma (2008)** | 20 years old | F | Recurrent chest infection, cough, low-grade fever. exacerbation of cough on ingestion of food/fluids |  | right (RLL) | pulmonary | an area of haziness with fluffy nodules in the right paracardiac zone | focal bronchiectasis at the medial basal segment. Multiple confluent acinar opacities | passage of barium into the bronchus of the right posteromedial basal pulmonary segment | confirmed the imaging findings |  | III | Segment resection | Constrast | 1 year， well |
| **Yutaka (2007)** | 50 years old | M | Right epigastric pain |  | right (RLL) | systematic |  | right-sided, enhanced mass, arterial branches coming from the aorta. |  | angiography: arteries from the aorta, the celiac artery, the right subphrenic artery |  | III | RLL lobectomy | intraoperative |  |
| **Eom（2007）** | 18 years | M | Dry cough, mild dyspnea | left pericardial defect | left | systematic |  | cystic mass with an air fluid level connected with esophagus in the middle mediastinum, left pericardial defect |  |  |  | III | Surgical excision of the mass | pathology |  |
| **Singal (2006)** | 15 months | F | Recurrent episodes of pneumonia and failure to thrive, frequent regurgitation |  | bilateral | systemic (both) |  | esophageal bronchi originating from the lower esophagus on both sides | two tubular structures originating from the lower esophagus and leading to intrathoracic entities bilaterally |  |  | III | left lower lobectomy；right sequestration resection | contrast | well |
| **Lee (2006)** | Newborn | M | RDS | PDA | right | systemic | opacification of the right hemithorax, mediastinal shift | bronchopulmonary sequestration, a bronchus connected the distal esophagus to the sequestration, pulmonary sling | esophageal bronchus |  |  | II | right pneumonectomy, pulmonary sling reimplantation, tracheobronchoplasty | contrast |  |
| **Linnane (2006)** | 5 monts | F | pyrexia, vomiting and coughing, RDS | absence of a right radius and thumb, dysplastic right ulna and an absent right kidney. VACTERL | right | pulmonary | aplasia of the right lung with compensatory emphysema of the left lung |  | right broncho-esophageal fistula |  |  | II | Right pneumonectomy | Contrast | 13 months, asmyptomatic |
| **Seguier-Lipszyc (2005)** | Newborn | M |  |  | left |  | Initial normal left lung. 2nd day: complete atelectasis of the left lung | Vertical left mainstem bronchus origin more cephalic than right side, Tracheal stenosis, close contact between the esophagus and the left main stem bronchus | oesophageal implantation of the left main stem bronchus |  |  | IA | reimplantation of the left bronchus | contact | 3 years,repeat dilatation |
| **Becker (2005)** | newborn | F | RDS and drooling, episodic choking and cough | VACTERL, patent foramen oval, PDA | right | pulmonary | initial: both lungs are normal; 1st day postop: opacification of the right hemithorax, mediastinal shift | collapsed, contrast-filled lung, right mainstem bronchus was absent. | CPBFM from right distal esophagus to the right lung |  |  | IA | resection | contrast |  |
|  |  | F | dyspnea |  | left | systemic | opacification of the left lung | hypoplastic left lung, extrapulmonary sequestration, left bronchial atresia, small airway in the hypoplastic lung that appeared to connect with the esophagus. | bronchial connection with the distal esophagus |  |  | II | excision of the left pulmonary sequestration | CT |  |
| **Tsugawa (2005)** | Newborn | F | RDS |  | right |  | coil-up sign, atelectasis of the right lung, |  | right main bronchus arising from the lower esophagus | Bronchography showed agenesis of the right main bronchus and stenosis of the lower trachea and left main bronchus |  | IA | reimplant | contrast | died during the operation |
|  | 2 months | F | recurrent aspiration pneumonia since birth, RDS | chromosomal aberration, agenesis of the corpus callosum and low-set ears; ASD, VSD, PDA, bilateral hypoplastic pulmonary arteries | right |  |  | long-segment tracheal stenosis, stenosis of the left main stem bronchus and right upper bronchus | right bronchus arising from the lower esophagus |  |  | IV | right upper lobectomy | Contrast | Mechanical Ventilation |
|  | 14 months | M | recurrent pneumonia of the left lung |  | left |  | atelectasis of the left lung and a consolidated mass in the left hemithorax |  | tubular communication between the lower esophagus and the mass lesion in the left hemithorax | congenital long-segment tracheal stenosis.The left bronchus was absent. |  | II | left pneumonectomy | Contrast | 5 years |
| **Lucaya (2003)** | Newborn |  | RDS |  | right |  | soft tissue mass in the right upper lobe with some air bronchograms | tubular air-filled structure arising from the trachea | esophageal bronchus |  |  | III |  | Contrast |  |
| **Tsuchiya (2003)** | Newborn | M | RDS |  | right | pulmonary | opaque right hemithorax, shift of mediastinal | hypoplastic lung tissue, right main-stem bronchus communication between the upper gastrointestinal tract and right anomalous lung tissue | communication between the esophagus and the right lung tissue | fistula at the lower part of the esophagu, atresia of the right main bronchus |  | II | right pneumonectomy | contrast, bronchoscopy | 1 year, stable |
| **Borsellino (2002)** | Newborn | F |  | diaphragmatic eventration | left | systemic | consolidation of the left lung base | pulmonary sequestration and a diaphragmatic hernia |  |  |  | III | resection, diaphragm plication | intraoperative findings | well |
| **Rahman(1999)** | 36 years old | F | shortness of breath, scant hemoptysis, cough |  | right |  | right paramediastinal cyst, and right-sided infiltrate | right lower lobe cyst containing multiple air fluid levels, with adjacent lung showing numerous cystic lesions, air space disease, and fibrosis | normal | hyperemic airways. | post ERCP: fibula connecting the main pancreatic duct with the right pleural space | IV | 1. right middle lobectomy and lower lobectomy; 2. distal pancreatectomy | ERCP | 6 months, uneventful |
| **Saydam (1999)** | Newborn | F | Cyanosis at birth | tetralogy of Fallot, PDA; DiGeorge syndrome | left |  | Aerated lung bilaterally. post, left lung collapsed |  |  | after ligation of TEF: Atretic left mainstem bronchus |  | IA |  | Bronchography, autopsy | died |
| **Sumner (1997)** | newborn | F | spitting copious amounts of mucus at birth. | vetebral anomalies, imperforate anus, ambiguous genitalia, annular pancrease. VACTERL | right |  | a small hypoaerated right lung，postoperative: right lung collapsed | absence of normal right mainstem bronchus, right mainstream bronchus originating from the distal esophagus | a branching tubular structure that extended from the distal third of the esophagus to the right lung |  |  | IA |  | contrast |  |
| **Srikanth (1992)** | newborn | F | RDS |  | right (RLL) | systemic | EA/TEF, opaque lung field |  |  |  | aortagram | IB | fistula ligation not resection | intraoperative findings | Died 3 weeks for infection |
|  | 5 months | F | RDS and poor feeding | fusion of upper ribs bilaterally | right |  | Opaque right lung, mediastinal shift to the right, main bronchus arising from the trachea |  | Right main bronchus arising from the lower esophagus |  |  | II | resection | contrast |  |
|  | 6 months | M | Breathlessness, choking upon feeding, recurrent pneumonias |  | right (RUL) |  | Right upper lobe mass |  | Fistula from the mass to the esophagus | the right upper lobe bronchus emanated from the trachea and the right main bronchus supplied the middle and lower lobes |  | III | right upper lobe with the mass and fistula were resected | intraoperative |  |
|  | 4 days | F | Bilious emesis | Malrotation | right | systemic | showed a right lower thoracic paravertebral mass |  | Fistula from the GE junction to the mass and malrotation |  | Aortogram showed a large feeding vessel from the thoracic aorta. | III | resection | Contrast |  |
|  | 2.5 months | F | Pneumonia |  | right | systemic | right lower lobe mass. |  | fistula from a lower esophageal diverticulum to the right lower lobe mass. | bilateral mainstem bronchial stenosis | aortogram | III | Resection | Contrast |  |
|  | 15 months | F | postprandial emesis and chronic nocturnal cough. | CDH | right | systemic | right lower lobe consolidation. |  | Thoracic herniation of the stomach and malrotation |  | Aortography | III | resection | intraoperative |  |
| **Michel (1997)** | newborn | F | Respiratory symtoms |  | right | pulmonary | Right lung opacity |  | Right bronchial tree connected to the esophagus | Absence of right main bronchus | Esophagoscopy: fistula at the lower third of the esophagus | II | Reimplant | contrast, bronchoscopy | Bronchomalacia, brooches stenosis, Reoperation after 1 years |
|  | Newborn | M | Slight RDS | PDA | left | pulmonary | mediastinal shift, atelectasis of the left lung | mediastinal shift, atelectasis of the left lung | main left bronchus connect to the esophagus |  | Angiography: normal pulmonary arterial supply and venous drainage, PDA | II | Reimplantation | Constrast | Left atelectasis, anastomotic stenosis |
| **Usui (1995)** | Newborn | F | RDS | Micrognathia, ankyloglossia, uranoschisis, bilateral low-set ears, hypoplasia of right external ear, ASD, cor triatriatum, PDA, | right | pulmonary | Opaque right hemithorax with right mediastinal shift |  | Right main bronchus arising from the lower part of the esophagus | Distal trachea stenotic, complete cartilage ring, lacked right main bronchus | Normal bilateral main pulmonary arteries | II | reconstructive operation | Contrast | ventilatory insufficiency, died postop day 5 |
| **Murray(1993)** | Newborn | M | RDS, worsen when naso-gastric feeding was attempted | Bilateral DDH, flexion defeormities of both knees, bilateral talipes calcaneovarus, fusion of L5 and S1, absence of two sacral segments; a duplication cyst in the third part of the duodenum, foregut duplication | bilateral | pulmonary | bilateral basal consolidation/collapse, left-sided mediastinal mass | Consolidation in both lower lobes | bilateral esophageal bronchi arising from the distal esophagus |  |  | III | No | Contrast | Died 107 days, jaundice, sepsis |
| **Jamieson (1993)** | newborn | M | Mild distress and drooling. |  | right (RLL) | systemic | initially lung were clear |  | postop: fistula from the distal esophagus to the posterior right lower lobe |  |  | III | resection | contrast | Tracheomalcia and GER |
|  | Newborn | M | Drooling | ARM, sacral deformity, ASD | right | systemic | lung was clear initially, postoperative consolidation in th right mid and lower lung |  | fistula from the distal esophagus to the opacified right lung |  |  | III | resection of middle and lower lobe | contrast | GER, tracheomalcia |
| **Matsumoto (1995)** | Newborn | M |  |  | right |  |  |  | Hypoplastic right lung arising from the distal esophagus. |  |  | IA | right pneumonectomy | contrast | pneumonectomy syndrome, respiratory distress, tacheomalcia, GER, died of sepsis and DIC at the age of 2 years |
|  | newborn | F |  |  | right |  |  | Hypoplastic right lung arising from the distal esophagus | Hypoplastic right lung arising from the distal esophagus |  |  | IA | right pneumonectomy | contrast | pneumonectomy syndrome |
| Yang(2018) | 6 months | F | RDS, fiver, dyspnea | pulmonary artery sling | right | pulmonary | opacification of right lung | right mainstem bonchus connected to esophagus | right main-stem bronchus arising from esophagus | Absence of right main bronchus |  | II | right pneumoectomy | contast, CT | 6 months, normal |
